# Single Fragment or Bulk Soil DNA Metabarcoding: Which is Better for Characterizing Biological Taxa Found in Surface Soils for Sample Separation?

**DOI:** 10.3390/genes10060431

**Published:** 2019-06-06

**Authors:** Laura M. Boggs, Melissa K. R. Scheible, Gustavo Machado, Kelly A. Meiklejohn

**Affiliations:** Department of Population Health and Pathobiology, North Carolina State University, 1060 William Moore Drive, Raleigh, NC 27607, USA

**Keywords:** DNA barcoding, DNA metabarcoding, plants, animals, forensic-type soils

## Abstract

In forensic geology casework, sample size typically limits routine characterization of material using bulk approaches. To address this, DNA-based characterization of biological taxa has received attention, as the taxa present can be useful for sample-to-sample comparisons and source attribution. In our initial work, low biodiversity was captured when DNA barcodes were Sanger-sequenced from plant and insect fragments isolated from 10 forensic-type surface soils. Considering some forensic laboratories now have access to massively parallel sequencing platforms, we assessed whether biological taxa present in the same surface soils could be better characterized using DNA metabarcoding. To achieve this, plant and animal barcodes were amplified and sequenced on an Illumina^®^ MiniSeq for three different DNA sample types (*n* = 50): individual fragments used in our initial study, and 250 and 100 mg of bulk soil (from the 10 sites used in the initial study). A total of 572 unique target barcode sequences passed quality filtering and were used in downstream statistical analyses: 54, 321, and 285 for individual fragments, 100 mg, and 250 mg bulk soil samples, respectively. Plant barcodes permitted some spatial separation of sample sites in non-metric multidimensional scaling plots; better separation was obtained for samples prepared from bulk soil. This study confirmed that bulk soil DNA metabarcoding is a better approach for characterizing biological taxa present in surface soils, which could supplement traditional geologic examinations.

## 1. Introduction

Geologic material, such as soil and dust, are ubiquitous in the environment and, as such, are important types of trace forensic evidence. For decades, highly trained forensic geologists have used various analytical approaches to characterize the inorganic components (i.e., minerals) of geologic material, with the information gleaned often used for sample-to-sample comparisons (e.g., does the soil from the suspect’s boot match that from the crime scene ?) [1]. A more challenging, laborious, and arguably more important use of forensic soil examinations is in the geographic attribution of evidence. These ‘provenance’ examinations can provide extremely useful information both for investigative leads or for broader intelligence gathering [2]. Geologic materials, especially those associated with provenance cases, can be extremely limited in sample sizes (i.e., <200 mg), derived from an unknown pedigree (e.g., is the debris from dust, top soil, or soil from a subsurface horizon?) and adhered to varied substrates (e.g., garments, shoes, shovels/trowels, tire/wheel wells). These characteristics make it difficult to characterize the inorganic components using routine bulk analyses. 

To supplement traditional geologic examinations, there has been a push to utilize the presence of organic components (i.e., biological taxa) for both sample-to-sample comparisons and geographic attribution, given direct associations can be made between organisms and their environment. Morphological characterization of pollen, diatoms, and foraminifera associated with geologic evidence has often been used in forensic investigations to permit discrimination between samples and provide distinct information on sample origin (e.g., [3,4,5,6,7]). Despite their utility, morphological characterization of these components is time-consuming, can only be performed by highly trained experts, and specifically for pollen, the taxonomic resolution attainable for a typical forensic sample is low (i.e., family/genus or higher) [4,6,8].

Thus, to fully harness the value of biological taxa for forensic geologic examinations, characterization using various DNA-based approaches have been explored. With advances in sequencing technologies (i.e., massively parallel sequencing (MPS)), much of the work in this arena has focused on DNA metabarcoding. This approach is an adaptation of the widely used DNA barcoding method that permits species-level identification of unknown taxa; instead of using Sanger technology to sequence the informative region(s) of the genome from individual biological taxa, using DNA metabarcoding, these region(s) are simultaneously sequenced from all biological taxa present in a bulk sample (e.g., water, soil, dust) on an MPS platform. DNA metabarcoding of soil is already widely used in ecology to accurately reconstruct past and present vertebrate and invertebrate biological communities (e.g., [9,10,11,12,13,14]). In a forensic context, the vast majority of published studies that have applied DNA metabarcoding to geologic materials have focused on characterizing the bacterial community, with some reporting that individualization is possible even amongst spatially similar samples [15,16,17,18,19]. 

While the sensitivity and high-throughput capabilities of DNA metabarcoding make this an attractive approach for characterizing diverse biological taxa (e.g., plants, animals, bacteria, fungi) from forensic geologic evidence, there are several hurdles restricting its broad implementation. Perhaps the most rudimentary of these lies in access to MPS instruments by forensic laboratories. While some larger state and federal forensic laboratories have or are currently validating MPS for traditional human DNA forensic analysis (i.e., short tandem repeats (STRs)), acquiring an MPS instrument is out of reach for most smaller laboratories, given their high cost. Considering this, we focused our initial efforts in this area on developing a modified protocol to obtain DNA barcode sequences from individual plant and insect fragments isolated from forensic-like biological materials using Sanger sequencing [20]. The rationale for this was three-fold: 1) Most forensic laboratories that complete traditional human DNA analysis have a genetic analyzer instrument for STR analysis, which can be easily adapted to permit Sanger sequencing; 2) Unlike MPS, the use of Sanger sequencing technology is not new for the criminal justice system, as it has been used for decades for human mitochondrial DNA analysis; 3) Plant and insect material associated with geologic evidence is not routinely examined by forensic geologists and, thus, consuming this material for DNA analysis does not impede other examinations (whereas DNA metabarcoding requires consumption of the geologic sample). Despite developing a protocol that could generate DNA barcode sequences from forensic-like biological materials, only low biodiversity was captured, resulting in poor discrimination between sample sites [20]. This result was possibly a reflection of the reduced sensitivity of Sanger sequencing, but also the approach employed; only 10 plant and 10 insect fragments from each surface soil sample were characterized using DNA barcoding [20].

Another challenge for any forensic analysis is the sensitivity and reproducibility of the results. It has been well reported that it is not possible to identify the exact same set of biological taxa through DNA metabarcoding across duplicate reactions (i.e., either from the same starting DNA extraction or different extractions from the same original bulk sample) [18,21,22]. This is due to the stochastic effects of PCR and variation in the bulk material used in DNA extractions, despite attempts to homogenize prior to subsampling. This poses a concern for the application of DNA metabarcoding to forensic casework as it could easily be argued by the defense that variation amongst evidence samples could be indicative that they did not originate from the same source. To address this, most DNA metabarcoding studies, at a minimum, perform duplicate PCR reactions from a single extract that are pooled prior to downstream processing (e.g., [18,21,22,23,24]). Additionally, some studies have implemented completing duplicate extractions for each sample (e.g., [21,22,23]), such that subsample variation can be adequately assessed. In a forensic scenario, however, where the amount of sample available for consumptive analysis is typically limited (i.e., <200 mg), this latter approach may not be feasible.

To address these hurdles, this study aimed to examine: 1) Whether increased discrimination amongst surface soils is possible from individual biological fragments, if the resulting DNA barcode amplicons are sequenced on an MPS platform; 2) Whether commonly employed strategies can improve the reproducibility of DNA metabarcoding from bulk soil, even when the amount of soil available for consumptive analysis is below the typical recommended amount (i.e., <250 mg); 3) Whether, through statistical analyses, the biological taxa identified in surface soils (either from individual fragment or bulk soil DNA metabarcoding) can spatially separate surface soils collected within Virginia, USA. 

## 2. Materials and Methods 

**Figure 1:**
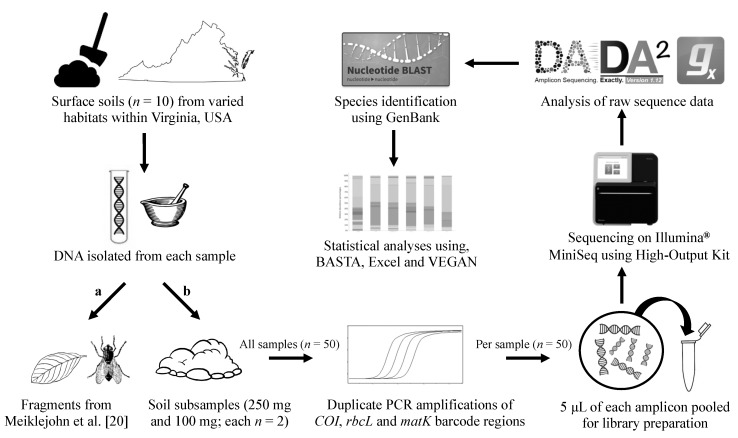
Schematic illustrating the major steps in the generation and analysis of DNA metabarcode data in this study: sample collection, DNA extraction, barcode region amplification, library preparation and sequencing, and sequence and statistical analyses.

The main steps followed to generate and analyze DNA metabarcode data in this study are shown in Figure 1. 

### 2.1. Soil Samples

Surface soils (0–3 cm) collected from 10 varied habitats within Virginia, USA, from October 2014 to May 2015, also used in Meiklejohn et al. [20], were used in this study (see Figure S1 for sampling locations). To mimic how geologic evidence is stored and processed, soils were allowed to air-dry at room temperature before storage in a laboratory environment in zip-lock plastic bags. Material was well homogenized (i.e., using a clean spatula to dissociate large clumps of material) before subsampling for subsequent DNA extractions.

### 2.2. Individual Fragment DNA Extractions and Pooling

The pre-isolated DNA from ~10 individual insect and plant fragments from each surface soil that was used in Meiklejohn et al. [20] was obtained (total *n* = 191). Briefly, the manufacturer’s protocols for the Qiagen DNeasy Plant Mini Kit (Qiagen, Hilden, Germany) and the Qiagen DNeasy Blood and Tissue DNA Purification Kit (Qiagen) were followed to extract DNA from both plant and insects. For each sample site (*n* = 10), separate plant and insect fragment DNA pools were created in Lo-Bind tubes (Eppendorf, Hamburg, Germany) by combining equal volumes of each individual DNA extract (e.g., 3 μL from each of the 10 plant fragment extracts from a given surface soil sample; Table S1). DNA extracts were stored at −20 °C when not in use. 

### 2.3. Bulk Soil DNA Extraction and Quantification

Total genomic DNA was extracted from dried surface soils using the DNeasy PowerSoil Kit (Qiagen). To assess the sensitivity and reproducibility of DNA metabarcoding, 4 extractions were completed for each sample site: 2 using the suggested input soil amount (~ 250 mg) and 2 using a lower amount (~ 100 mg) (see Table S2 for specific dry soil input weights for each extraction). The later extractions were incorporated to reflect what might be available for consumptive processing in forensic casework. At most 16 samples were extracted concurrently, and a reagent blank was processed with every batch. The manufacturer’s protocol was followed with one exception: the DNA was eluted into two eluates of 50 µL of Solution C6 as opposed to one eluate of 100 µL, to increase the final DNA concentration (see Table S1 for details). The quantity of DNA (ng/μL) extracted was assessed using the Qubit™ 3 Fluorometer (Invitrogen, Carlsbad, CA, USA) and the Qubit™ dsDNA High Sensitivity (HS) Assay Kit (Invitrogen). Every reagent blank was verified as clean via the Qubit™ (i.e., no reading observed), and thus not carried through downstream processing. DNA extracts were stored at −20 °C when not in use.

### 2.4. Amplification and Quantification

All amplifications were performed on a Veriti™ 96-well Thermal Cycler (Applied Biosystems™, Foster City, CA, USA). To allow direct comparison with the results obtained in Meiklejohn et al. [20], the same traditional barcoding loci were targeted: cytochrome oxidase subunit I (*COI*) for animals, and maturase K (*matK*) and ribulose biphosphate carboxylase (*rbcL*) for plants. While alternative barcode loci that permit discrimination and can be more straightforwardly sequenced on MPS platforms have been identified and assessed (e.g., *trnL*, *ITS2*, and *psbA-trnH* in plants [25,26,27,28,29,30,31,32,33,34]), these were not targeted in this study. For clarity, amplifications of plant barcoding loci *matK* and *rbcL* were completed using the KAPA3G Plant DNA polymerase (KAPA Biosystems, Wilmington, MA, USA) and the *COI* barcode region was amplified using the Q5^®^ Hot Start High-Fidelity DNA polymerase (New England BioLabs Inc, Ipswich, MA, USA). It is important to emphasize that while the *COI* primers used in Meiklejohn et al. [20] amplified the appropriate region from individual insect fragments, these primer pairs are universal and contain some degenerate bases at the third codon position to permit successful amplification across a diverse range of vertebrate and invertebrate animal taxa [35,36,37,38,39,40]. A negative and appropriate positive control (either an extract of a *Gerbera* sp. or *Vespula* sp. for plants and animals, respectively) were included in each PCR. Each target was amplified in duplicate to increase the likelihood of adequately capturing species richness [41] and to reduce the impact of PCR stochasticity that is often observed when the DNA template is of low quantity or quality (e.g., [42,43,44]). PCR was completed in six batches; one batch per target, in which duplicate amplifications for all samples were processed. Table 1 outlines the primers used and amplifications completed on each sample type. To determine amplicon quantity, 5 µL from each duplicate reaction was combined, purified with 18 µL (1.8×) of Agencourt AMPure XP Reagent (Beckman Coulter, Brea, CA, USA), and quantified using the Qubit™ 3 Fluorometer (Invitrogen) and the Qubit™ dsDNA HS Assay Kit (Invitrogen). A subset of purified amplicons, representing all sample types and sites, were visualized via gel electrophoresis using the Lonza FlashGel® System (Lonza, Rockland, ME, USA) and 2.2% agarose FlashGel^®^ DNA cassettes (Lonza). 

### 2.5. Library Preparation

For each sample (*n* = 50) along with a positive and negative control, a single library was prepared and consisted of DNA from each of the six barcode regions amplified in duplicate (*n* = 12 amplicons). In a single well of a 96-well plate, 5 μL of each amplicon were combined creating a total volume of 60 μL per sample. To remove potential primer dimer prior to library preparation, each 60 μL amplicon pool was purified with 108 μL (1.8×) of Agencourt AMPure XP Reagent (Beckman Coulter), and subsequently eluted in 60 μL of Buffer EB (Qiagen). A total of 50 μL of each purified amplicon pool was used as input for library preparation using the KAPA Hyper Prep Kit (KAPA Biosystems). Following the manufacturer’s protocol, libraries were prepared with compatible Illumina^®^ indices/adapters and eluted in 25 μL of elution buffer following purification. Each library (*n* = 52) was individually quantified using the KAPA Library Quantification Kit (KAPA Biosystems), and combined in a single LoBind tube (Eppendorf) to create an approximately equimolar library pool. The concentration of the final library pool was verified using the KAPA Library Quantification Kit (KAPA Biosystems). 

### 2.6. Sequencing Using the Illumina^®^ MiniSeq

The pooled library was prepared as described in the Denature and Dilute Libraries Guide (Document # 15039740 v10) with a 50% PhiX spike and final loading concentration of 1.4 pM. This pool was subsequently sequenced on a single run of the Illumina^®^ MiniSeq using the MiniSeq System High-Output Kit (Illumina, San Diego, CA, USA). The MiniSeq was chosen for use in this study over the Illumina^®^ MiSeq for several reasons: 1) The MiniSeq provides a better entry point into the MPS market for smaller forensic laboratories, due to a substantially lower instrument cost; 2) The sequencing time is nearly halved for a similar type of run on the MiniSeq (~24 vs. 48 h), which could prove vital in an expedited forensic case; 3) Output and read quality are not compromised, being highly comparable between platforms (both ~22 million single-end and 44 million paired-end reads). One limitation of the MiniSeq however, is that it can generate at most 150 bp paired-end reads (i.e., 150 bp from both the 5’ and 3’ end of an amplicon) or a single 300 bp read (i.e., 300 bp from either the 5’ or 3’ end of an amplicon, as the indices/adapters randomly attach to one end of an amplicon). Given the length of four out of the six barcode amplicons are >590 bp (Table 1), if paired-end sequencing was completed on the MiniSeq, associations between reads derived from the same amplicon would not have been possible; a gap of 300–550 bp would have prevented the straightforward alignment of read pairs as it relies on some matching overlapping sequence. Thus, we decided to complete the sequencing using the 1 × 300 bp conformation to obtain the most amount of data from a single amplicon. While 300 bp is shorter than the average length of most barcode sequences obtained in Meiklejohn et al. [20] (297, 532, and 409 bp for *COI*, *matK*, and *rbcL*, respectively), studies encompassing a diverse range of taxa and barcode regions (e.g., *COI*, fishes, butterflies, wasps, flies, carnivores [49,50,51,52]; *rbcL*, land plants [48]) have shown that there is limited negative influence on the discrimination power when shorter portions of a barcode region are sequenced. 

### 2.7. Processing Massively Parallel Sequencing Data

A bioinformatics pipeline was specifically developed for processing the barcode sequence data generated in this study. Raw reads contained in the FASTQ files generated from the sequencing run were filtered and trimmed using the default parameters of the DADA2 pipeline [53]. After the removal of any chimeric sequences, the resulting reads were denoised and only unique sequences (also known as exact sequence variants) retained. The DADA2 pipeline produced two files: 1) A csv file, which listed each unique sequence string, the sequence ID code assigned to each of these sequences and the count of each sequence per sample; 2) A FASTA file, which contained each unique sequence labeled with the corresponding sequence ID code, which was imported into CLC Genomics Workbench v11.0.1 (CLC; Qiagen, Aarhus, Denmark). To identify the primer pair used to generate each unique sequence, the default settings of the “finding binding sites” and “create fragments tool” in CLC was run separately for each primer (*n* = 12). The output from these tools was a list of sequences which contain the specific searched primer sequence. Using those lists, 12 separate and specific FASTA files were created using the “seqtk-subseq tool” on the Galaxy web platform (usegalaxy.org; [54]), that only included sequences that matched a single primer. For each separate FASTA file, the appropriate primer sequence was subsequently trimmed from either the 5’ or 3’ end using the “trim adapter” functionality in CLC. Given some primers are nested within the target region of another, it was possible that more than one primer sequence could have been identified in the same sequence (e.g., the *COI* mini primers are nested within the *COI* entire primers, thus a sequence could contain the LCO1490-L sequence and the uniminibarF1 sequence). In this scenario, that sequence read would have been represented in more than one primer subset. To remove these ‘duplicates’, only sequences of the expected length were retained for each primer. For example, *COI* mini is only a 130 bp amplicon and, thus, if a sequence >130 bp appeared in the FASTA file for uniminibarF1, that sequence would be discarded as it must have originated from LCO1490-L. 

### 2.8. Searching Sequence Data against GenBank

The filtered and trimmed unique sequences were searched against GenBank’s nucleotide database, BLASTN (https://blast.ncbi.nlm.nih.gov/Blast.cgi), using the remote command line interface. To streamline the classification of matches to higher taxonomic groups, the taxonomy database commands were enabled during BLASTN searches. The default BLASTN settings were used, and the top 10 matches (based on expect value (E-value)) were written to a tsv file. The GenBank ‘taxid’ output for each match was subsequently used as input into the taxize program [55] to retrieve more detailed taxonomic information (e.g., class, order, family, etc.) for use in biodiversity comparisons and statistical analyses. To ensure downstream statistical analyses excluded non-target sequences (e.g., those derived from possible contaminants or non-specific amplifications), unique sequences were filtered to only include those that matched the expected broader taxonomic group (i.e., *matK* and *rbcL* sequences to the plant kingdom and *COI* sequences to animal kingdom, referred to herein as unique target sequences). Microsoft^®^ Excel for Mac v16.16.1 (Microsoft, Redmond, WA, USA) was used for parsing and comparing the BLASTN results. 

Our initial intention in the statistical analyses was to only include unique target sequences that met specific BLASTN criteria (i.e., those commonly enforced in DNA metabarcoding studies to assign taxonomy): 1) A minimum of 90% of the query sequence present in the subject sequence returned from the BLASTN search (i.e., sequence coverage); 2) At least 95% of overlapping nucleotides being identical (i.e., sequence identity); 3) The match had an E-value of less than 0.001 [27,29,33]. Only 36 out of the 1293 unique target sequences met these criteria, with most only present in samples prepared with the pooled individual fragment DNA. The inclusion of all 1293 unique target sequences in downstream analyses was problematic, given that: 1) The BLASTN matching statistics, especially E-value and percent coverage, were suboptimal on average; 2) Over 50% of sequences returned multiple records with the same best match statistics, which were incongruent at the genus or species level (Table S3). Thus, we used the BAsic Sequence Taxonomy Annotation tool (BASTA; [56]) to identify the last common ancestor for unique target sequences in which the best match(es) had an alignment length of >25 nucleotides, 85% sequence identity, and E-value of <1. This subset of unique target sequences formed the final dataset for downstream analysis.

### 2.9. Data Analysis

Similar to previous studies (e.g., 9,11,15), two different approaches were used to visualize the DNA metabarcoding data: 1) Taxon abundance charts were generated in Microsoft^®^ Excel for Mac v16.16.1 (Microsoft) for each sample type and sample site, to visualize variation in taxa presence and relative abundance; 2) Non-metric multidimensional scaling (NMDS) plots were generated using the R (v 3.5.1) package VEGAN (v 2.5.3) at default configuration [57] and visualized in ggplot2 (v 3.1.1) [58] to assess whether spatial separation between samples sites was present using taxa that were identified using DNA metabarcoding. 

To generate the taxon abundance charts, the abundance (i.e., number of reads) for each of the 10 Eukaryote phyla identified in the BASTA analysis was calculated for each sample (*n* = 50): Annelida (e.g., worms), Arthropods (e.g., insects), Cercozoa (e.g., algae), Chordata (e.g., mammals, reptiles), Cnidaria (e.g., jellyfish, coral), Mollusca (e.g., mollusks), Nematoda (e.g., nematodes), Porifera (e.g., sponges), Rotifera (e.g., microscopic aquatic animals), and Streptophyta (e.g., plants). The abundance of each phyla per sample type and sample site was calculated and plotted (in some plots duplicates for the bulk soil samples were summed).

Non-metric multidimensional plots have been successfully used in previous studies to spatially separate samples [15,17,21]; bacteria present in top-soil samples permitted clustering of samples by habitat, regardless of age and storage conditions [15]. While these plots may appear simplistic, they could be a valuable visual aid in the courtroom setting; jurors could see whether the evidence and known sample cluster together in multidimensional space, as opposed to trying to interpret the relative proportions of a set of species (or pertinent taxonomic group) recovered between the two samples. To generate the NMDS plots in this study, five separate matrices were created (all sequences, only *COI*, only *matK*, only *rbcL*, and combined *rbcL* and *matK*), which for each sample (*n* = 50) contained only the sequences meeting the thresholds in the BASTA analysis. The matrix was not binary; rather, it included the total number of reads recovered for that sequence in a given sample as a proxy for taxa abundance. Kruskal’s stress was measured for each plot and two dimensions were used for all NMDS plots, given that additional dimensions did not substantially improve the resolution. To facilitate visualization and comparison across plots, samples originating from the same site were plotted in the same color (e.g., all five samples from Site M are shown in pink) and sample types denoted by different shapes (i.e., 250 mg bulk soil—triangle, 100 mg bulk soil—circle, pooled individual fragments—square).

## 3. Results and Discussion

### 3.1. Total Genomic DNA Yields from Bulk Soil

From each soil sample (n = 10), the total genomic DNA was extracted in duplicate using both the suggested input soil amount and an amount lower than optimal (total n = 40). No significant difference (t-test, p = 0.0525) in total yield was observed between extracts completed using 250 mg (1.48 ± 1.27 μg) and 100 mg (0.84 ± 0.57 μg) of soil (each n = 20). This finding is important as having ≥250 mg of soil available for consumptive processing is unlikely in a forensic scenario. This result does conflict with that reported by Young et al. [21], in which a significant decrease in DNA yield was observed when suboptimal amounts of soil (i.e., 150 mg and 50 mg) were used as input. Young et al. [21] did, however, also report that a decrease DNA yield did not negatively impact the number of subsequently recovered operational taxonomic units (i.e., effectively unique taxa). Despite homogenizing the soil prior to subsampling, significant variation in the yield among duplicate 250 mg extracts was observed (t-test, p = 0.0024). However, a similar finding was not observed when 100 mg was used (t-test, p = 0.9301) (Table S2). Additionally, only when 250 mg of soil was used as input did the density of the soil significantly impact DNA yield; sites in which the bulk density was indicative of a soil rich in organic matter (~ < 0.6 g/cm^3^; Table S2) had significantly lower DNA yields (t-test, p = 0.007). 

### 3.2. Polymerase Chain Reaction Amplification

**Figure 2:**
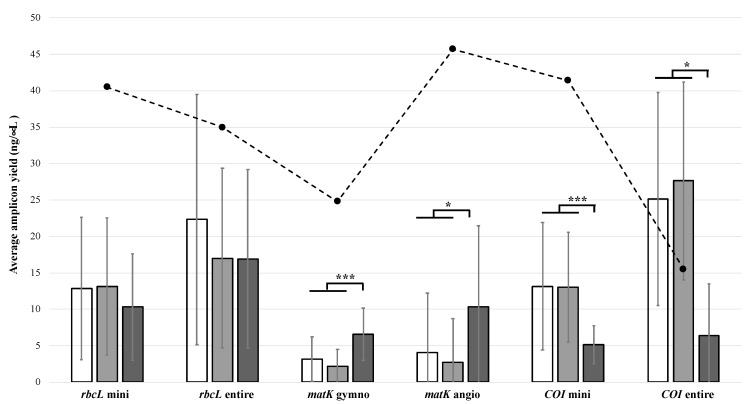
Amplification success of DNA barcode regions using six different primer pairs. Average (± standard deviation) amplicon yield (ng/µL) across all samples prepared using 250 mg of bulk soil (*n* = 20; denoted by white bars), 100 mg of bulk soil (*n* = 20; denoted by light grey bars) and individual fragments (*n* = 10; denoted by dark grey bars). Significant differences in yield (as determined by *t*-test) are shown, with * denoting *p* < 0.05 and *** denoting *p* < 0.001. The dashed line represents the yield of the positive control.

Using the same PCR reaction mix constituents and cycling conditions optimized in Meiklejohn et al. [20], amplification with the six primer pairs was successful in this study across all sample types. After purification, amplicons of the expected size were observed from both the bulk soil and pooled individual fragment DNAs via agarose gel electrophoresis. For each of the six amplicons, no statistical difference in amplicon yield was observed between extracts prepared with 250 mg and 100 mg of soil (*t*-test, *p* = 0.2514–0.9672). When comparing yields from all bulk soil extracts to those obtained from pooled DNA extracted from individual fragments, a significant difference in yield was observed for four out of six amplicons (Figure 2). For both *matK* amplicons, higher yields were consistently obtained from the pooled individual fragment DNAs (Figure 2, Table S1, Table S2). Given the known difficulties with amplifying *matK* due to size (~850 bp) and lack of robust universal primers, it is not surprising that DNA derived from intact individual plant fragments yielded better results. In contrast to this, higher yields for both *COI* amplicons were obtained from the bulk soil extracts (Figure 2). The *COI* primers used in this study are universal and have been widely used in studies with diverse invertebrate and vertebrate animal taxa [35,36,37,38,39,40]. In our initial study [20], the total quantity of DNA isolated from individual insect fragments was low (0.45 ± 1.27 μg) and successful amplification of either *COI* region (i.e., entire or mini) was only obtained in 74% of fragments. Knowing this and that bulk soil extracts likely contain DNA from a range of animal taxa (not exclusively insects), obtaining higher yields from the bulk soil was anticipated. 

### 3.3. Barcode Sequence Recovery

Across 52 samples (including a positive and negative control), a total of 4,733,057 raw indexed reads were obtained from the single MiniSeq sequencing run. The range in the number of raw reads obtained for each sample type were as follows: 250 mg of bulk soil, 3798–125,068; 100 mg of bulk soil, 29,456–481,604; and pooled individual fragment DNA, 62,918–171,158. After processing the raw reads through DADA2, a total of 3,922,428 reads representing 15,587 unique sequences were identified. To group these unique sequences by primer pair, we processed the sequences as outlined in the Materials and Methods. Both the forward and reverse primer sequences were present in close to 85% of unique sequences. Given 1 × 300 bp sequencing was completed on the MiniSeq, both primer sequences should not be present in a single sequence for amplicons longer than 300 bp (i.e., *rbcL* entire, *matK* gymno, *matK* angio, and *COI* entire). While we observed relatively clean amplicons on agarose gels, we hypothesized that these sequences resulted from non-specific short amplicons that remained after bead-based purification. To confirm this, we subjected a subset of these sequences to a BLASTN search, in which they almost always matched to a bacterial, fungal, or environmental sample (albeit with poor matching statistics). Thus, these sequences were excluded and a total of 2375 unique sequences (represented by 479,386 reads) were used in downstream data processing. Optimization of the wet lab protocol to improve the recovery efficiency of ‘real’ barcode sequences was not completed in this study as a) by using the same conditions from our initial study [20], the impact of only modifying the sequencing approach could be assessed, and b) the protocol used was not too dissimilar from other DNA metabarcoding studies using soil (e.g., [15,17,21,22,23]). Given the small size of these erroneous sequences (majority <120 bp) and their biological origin, it is plausible that increasing the annealing temperature during PCR and implementing a second bead purification step would greatly improve the recovery efficiency of ‘real’ barcode sequences. 

### 3.4. Unique and Target Sequences

The total distribution of the 2375 unique sequences across the six barcode amplicons were as follows: *rbcL* mini, 471; *rbcL* entire, 472; *matK* gymno, 104; *matK* angio, 21; *COI* mini, 1248; and *COI* entire, 59. For all six amplicons, no significant difference was observed in the average number of unique sequences recovered between samples prepared with 250 and 100 mg of bulk soil (*t*-test, *p* = 0.1379–0.9813; Figure S2). However, for *rbcL* mini, *rbcL* entire, *matK* gymno, and *COI* mini, samples prepared with DNA isolated from individual fragments had, on average, significantly fewer unique sequences in comparison to those prepared from bulk soil (either 250 or 100 mg) (*t*-test, *p* = 0.0007–0.0079; Figure S2). 

While examining the number of unique sequences generated per amplicon provides useful information, only sequences that match the expected taxonomic group should be included for downstream sample characterization. In this study, only 1293 unique sequences (represented by 179,083 reads) were also classified as unique target sequences (see Materials and Methods for inclusion criteria). The majority of non-target unique sequences (*n* = 1083) were assigned to bacteria (α-, β- and δ-proteobacteria, high GC gram positive), algae (red, green, brown), and fungi. Given the specificity of the starting material varied, we observed, as expected, a higher proportion of unique target sequences from samples prepared with DNA isolated from individual fragments than those prepared with bulk soil (88.3 ± 17.4% and 54.3 ± 26.5%, respectively). Based on this result, we also expected the percentage of unique target sequences to be lower for the universal primer pairs than for the group-specific *matK* primers (Table 1). We mostly observed this trend with 63.8 ± 17.7% of unique sequences classified as unique target sequences across the four universal primer pairs. However, stark differences between the group-specific *matK* primer pairs were observed: 40.7% and 98.1% classified for *matK* gymno and *matK* angio, respectively. On a per-sample-type basis, significantly higher numbers of *rbcL* mini, *rbcL* entire, and *COI* mini unique target sequences were observed for bulk soil samples over those prepared from individual fragments (*t*-test, *p* = 0.0027–0.0391). 

In this study, a total of 572 unique target sequences met the BASTA inclusion criteria (see 2.8) and formed the final dataset for the statistical analyses. The distribution of these sequences were as follows: *rbcL* mini, 117; *rbcL* entire, 162; *matK* gymno, 2; *matK* angio, 8; *COI* mini, 275; and *COI* entire, 8. A total of 54, 321, and 285 of these unique target sequences were recovered from individual fragment, and 100 and 250 mg bulk soil samples, respectively. Both the individual unique target sequences (*n* = 572), and a matrix providing the number of reads for each unique target sequence per sample, are available via FigShare (10.6084/m9.figshare.8219039 and 10.6084/m9.figshare.8218997, respectively). 

### 3.5. Comparing DNA Barcoding to DNA Metabarcoding

DNA metabarcoding is extensively used in ecology as an approach to characterize and document biodiversity; species recovered from DNA metabarcoding are highly congruent with those identified using traditional survey approaches (e.g., [59,60,61,62]). While it is widely perceived that DNA metabarcoding permits higher sensitivity when compared to DNA barcoding, few studies have completed a side-by-side comparison of the impact of only implementing a different sequencing approach on the captured biodiversity. The design of this study allowed us to assess whether more information can be gleaned when sequencing DNA barcode amplicons via MPS as opposed to Sanger. In our initial study, the level of biodiversity captured when sequencing barcode amplicons using Sanger technology was low; 70% of recovered *rbcL* sequences (*n* = 76) were assigned to either Pinaceae (pine) or Fagaceae (oak/stone oak), and only 13% of *COI* sequences (*n* = 48) matched to the class Insecta [20]. In this study, a total of 54 unique target sequences that met the BASTA inclusion criteria were recovered across all samples prepared with DNA isolated from individual fragments. While sequencing barcode amplicons using MPS increased the biodiversity captured, 100% concordance was not obtained (Table S4, Table S5). For example, six different plant orders were identified from Site C using Sanger, with only one of these identified using MPS. Conversely, animal diversity from Sites F, H, and K was only captured when amplicons were sequenced using MPS. The *COI* sequences (*n* = 25) were primarily classified to either the Arthropoda (36%, exclusively insects) or Chordata (44%, exclusively mammals) phyla (Table S5). For the combined *rbcL* and *matK* sequences (*n* = 29; all Streptophyta), 52% were assigned to the class Magnoliopsida (flowering plants, mustards and allies, oak/stone oak and birch trees, grapes), 17% to the class Pinopsida (pines), 10% to the class Liliopsida (grasses/reeds), and 3% to the class Bryopsida (mosses) (the remaining 18% could not reliably be classified to a class using BASTA; Table S4). In general, the plant taxa recovered using DNA metabarcoding was reflective of the known land cover (Figure S1); pines are common throughout Virginia and were recovered from 80% of sites, and the common reed was recovered using both approaches only from the single coastal site (Site E; Table S4). The ability to amplify and sequence DNA regardless of the technology or approach relies, first and foremost, on template DNA being available. In general, the DNA extracted from individual plant and insect fragments was of low quantity and quality [20]. While we did observe an increase in biodiversity when using MPS, the success was likely limited by the template DNA and, also, the overall efficiency of the wet lab protocol (i.e., with respect to the recovery of ‘real’ barcode sequences). Greater biodiversity, especially for plants, was captured when DNA metabarcoding was completed using bulk soil (250 or 100 mg). 

### 3.6. Taxon Abundance and Utility for Sample Site Discrimination

**Figure 3:**
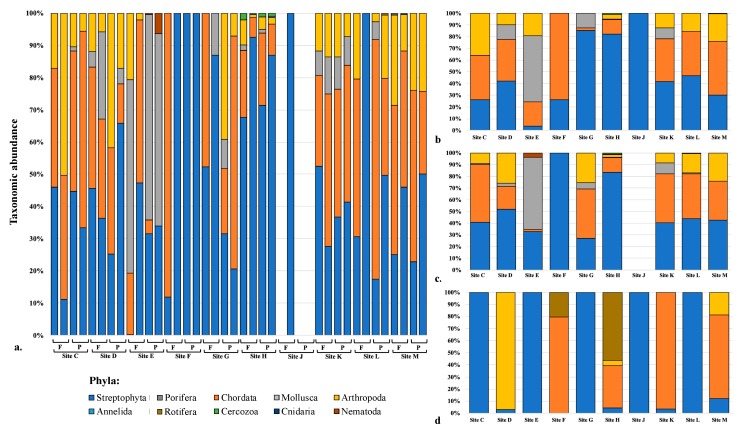
Relative abundance of 10 phyla recovered from samples collected from 10 sites across Virginia, USA. (**a**) Comparison between duplicate samples prepared with 250 mg of bulk soil (full—F) or 100 mg of bulk soil (partial—P). (**b**) Sum for samples prepared in duplicate with 250 mg of bulk soil. (**c**) Sum for samples prepared in duplicate with 100 mg of bulk soil. (**d**) Singularly from pooled individual plant and insect fragments.

Reproducibility is a well-documented issue with DNA metabarcoding, metagenomics, and microbiome analyses, in which the taxa recovered and their relative abundance can vary between duplicate samples. In disciplines such as forensics, where the taxa identified could provide valuable information for discriminating between samples, reproducibility needs to be adequately characterized prior to validation for casework. To preliminarily address this, we completed duplicate extractions using both 250 and 100 mg of bulk soil, and separately processed the DNA though the metabarcoding workflow (Figure 1). Similarly to previous studies (e.g., [15,21,23]), for each sample (*n* = 40), a) the unique target sequences that met the BASTA inclusion criteria (*n* = 572) were classified to a broad taxonomic group (i.e., one of 10 phyla in this study), and b) the overall abundance per group (i.e., number of reads) calculated and plotted (Figure 3a). The abundance of unique target sequences varied between duplicates (Table S6) and likely impacted the variation observed in the resulting taxon abundance chart (Figure 3a); the average difference in the number of assigned reads recovered between all duplicate samples, irrespective of input amount, was 44% ± 28% while the range was 8.8–100% (Table S6). For some sites, the variation was more pronounced; 250 mg samples from Site G were comprised of Streptophyta and either Chordata or Mollusca. This variation did, however, appear to correlate with the amount of DNA yielded from extractions (Table S2) and also the abundance and number of unique sequences recovered (Table S6, Table S7). Such variation between duplicates has been observed in other studies when the same parent sample is stored differently prior to processing or processed at vastly different times post-collection [15]. 

Combining the data from duplicate samples for analysis and comparisons could reduce variability but also improve the resolution between samples. When taking this approach in this study, clear distinctions between the 10 sample sites were still difficult to discern (Figure 3b,c). While for some of the sites, the taxon abundance charts generated using 250 and 100 mg were comparable (e.g., Sites H, K, L, M; Figure 3b,c), those from the individual fragment samples more often than not appeared markedly different (Figure 3d). Further, from our results it was not possible to establish an optimal sample type that could reliably permit site discrimination via taxon abundance charts (Figure 3b–d). Despite this, three main trends were noted: 1) Using bulk soil, Streptophyta and Chordata were the main phyla recovered at each site; 2) The highest proportion of Mollusca sequences were recovered from the single coastal site (Site E); 3) The composition of Site J was not diverse (i.e., only sequences from Streptophyta were recovered). While it could be argued that grouping sequences at lower taxonomic levels (e.g., class, family) could permit better discrimination between sample sites, this approach was not taken in this study as a) interpretation of such a chart would be difficult in a courtroom scenario, given large numbers of groups would be plotted (i.e., easily >100), and b) 38% of sequences in this study would have been excluded from the analyses, as the lowest common ancestor reported from BASTA for all unique target sequences (*n* = 572) was phylum. 

### 3.7. Using Non-Metric Multidimensional Scaling Plots to Spatially Separate Sample Sites 

**Figure 4:**
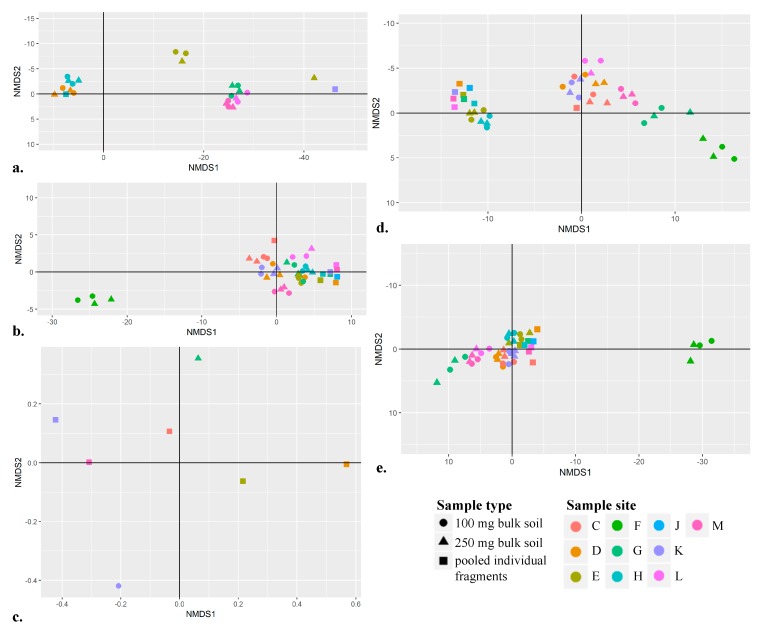
Non-metric multidimensional scaling (NMDS) plots of animal and plant taxa recovered from samples prepared with DNA isolated from 250 mg of bulk soil (triangle), 100 mg of bulk soil (circle), and pooled individual plant and insect fragments (square). The color of the shape denotes sample site (*n* = 10). Not all samples are visible (*n* = 50) as they either had no data for that marker (e.g., *matK*) or had extreme “x-axis limit” values and so were filtered out, or are plotted on top of one another in multidimensional space. Unique target sequences used to generate each NMDS plot are as follows: (**a**) *COI* entire and *COI* mini (*n* = 283), (**b**) *rb*cL mini and *rbcL* entire (*n* = 279), (**c**) *matK* gymno and *matK* angio (*n* = 10), (**d**) *rbcL* and *matK* (*n* = 289), (**e**) all *COI*, *rbcL* and *matK* (*n* = 572).

In this study, we created five NMDS plots with varied subsets of data: only *COI*, only *rbcL*, only *matK*, combined *rbcL* and *matK*, and all sequences (Figure 4a–e). When examining the site separation possible for each barcoding locus individually (Figure 4a–c), both *COI* and *rbcL* permitted some spatial separation in multidimensional space (Figure 4a,b). No discernable separation between sample sites was achieved when all 572 unique target sequences that met the BASTA analysis inclusion criteria were used in the analysis (Figure 4e). The most evident visual clustering of samples from the same site prepared using different approaches was observed when *rbcL* and *matK* data were used to generate the NMDS plot (Figure 4d). While there is some overlap in multidimensional space, clustering of samples derived from a single site is still apparent using *rbcL* and *matK*. This overlap (also noted in other NMDS plots; Figure 4) is likely not indicative of false positives, rather that the biodiversity recovered was the same for multiple samples (i.e., the same unique target sequences were recovered for more than one sample). Many of the samples prepared using DNA from individual plant and insect fragments clustered together multidimensional space (Figure 4b,d,e), rather than clustering with samples prepared from bulk soil from the same site. This result is likely a reflection of the similarity and reduced number of unique target sequences recovered from this sample type (*n* = 54). From these NMDS plots, two main trends were observed: 1) The plant barcoding loci, when combined, permit better spatial separation between sample sites compared to *COI*; 2) Better spatial separation is possible using data generated from bulk soil, given more unique target sequences (that represent varied taxa) are available for analysis. 

## 5. Conclusions

This study demonstrated that biological taxa in surface soils are better characterized when bulk soil DNA metabarcoding is completed over traditional DNA barcoding. Given many forensic laboratories are acquiring MPS instrumentation and expertise for traditional human DNA analysis, DNA metabarcoding of geologic evidence may not only be feasible but could be used to supplement traditional geologic examinations. However, before such a technique could be implemented into casework, a well-optimized and robust DNA metabarcoding protocol needs to be established. Based on the challenges encountered in this study with recovering ‘real’ barcode sequences for *COI*, *rbcL*, and *matK*, optimization should be focused on alternate barcode markers which are already widely used and more straightforwardly sequenced on MPS platforms (e.g., *ITS2*, *trnL*, and *psbA-trnH* for plants). No significant difference in DNA yield or the number of recovered unique target sequences was observed between extractions completed using optimal (250 mg) and suboptimal (100 mg) input amounts of bulk soil. Thus, using 100 mg as input in future DNA metabarcoding experiments not only seems prudent but also more applicable in a forensic context where limiting consumptive analyses is preferred. While the utility of taxon abundance charts for sample site discrimination in this study was limited, using plant barcodes recovered from bulk soil permitted some sample site separation in multidimensional space. Despite previous studies reporting promise for these approaches (e.g., [15,17,21]), the applicability to a courtroom scenario remains undemonstrated. Thus, future DNA metabarcoding studies should additionally compare the value of alternative statistical approaches (e.g., spatiotemporal regression analyses) for sample site separation, and establish an effective way to communicate the underlying statistical principles to a lay audience. Finally, the sample sites in this study were not only limited in number (*n* = 10) and geographic location (i.e., only Virginia, USA), but also spatial and temporal variation. Given that previously published studies have identified that biological taxa can vary within a sample site (e.g., 10, 50, and 100 m apart), between seasons and across soil depths (e.g., [16,23,27,63,64]), additional studies designed to comprehensively assess the impact of these variables on sample separation is warranted.

## Figures and Tables

**Table 1:** Barcoding loci amplified from pooled fragment (plant and insect) and bulk soil DNA. Primer pairs used per region are given.

Targeted Barcode Region and Primer Pairs (~amplicon length)	Pooled Plant Fragments (*n* = 10)	Pooled Insect Fragments (*n* = 10)	Bulk Soil (*n* = 40)
*matK*			
- matK-KIM-1R/matK-KIM-3F (850 bp) [45]	✓	✕	✓
- GymF1A/GymR1A (850 bp) [46]	✓	✕	✓
*rbcL*			
- rbcLa-F [47] /rbcLa-R [45] (590 bp) ^*^	✓	✕	✓
- rbcL1/rbcLB (230 bp) [48] ^*^	✓	✕	✓
*COI*			
- LCO1490-L/HCO2198-L (658 bp) [36] ^*^	✕	✓	✓
- uniminibarF1/uniminibarR1 (130 bp) [35] ^*^	✕	✓	✓

* denotes that the primer pair is universal.

## Supplementary Materials

The following are available online at https://www.mdpi.com/2073-4425/10/6/431/s1, Table S1: Results summary of the wet lab DNA metabarcode protocol for DNA isolated from individual insect and plant fragments collected at 10 sites within Virginia, USA. Table S2: Results summary of the wet lab DNA metabarcode protocol for DNA isolated from bulk soil (either ~250 or ~100 mg) collected at 10 sites within Virginia, USA. Table S3: Average (± standard deviation) BLASTN match statistics for the best match of each unique target sequence (*n* = 1293). Table S4: Comparison of plant taxa identified from each sample site when using DNA barcoding (i.e., Sanger) and DNA metabarcoding (i.e., MPS). Table S5: Comparison of animal taxa identified from each sample site when using DNA barcoding (i.e., Sanger) and DNA metabarcoding (i.e., MPS). Table S6: Abundance (i.e., number of reads) of unique target sequences which met the BASTA inclusion criteria (*n* = 572) across 10 phyla, for samples prepared in duplicate using 250 and 100 mg of bulk soil. Table S7: Number of unique target sequences which met the BASTA inclusion criteria (*n* = 572) across 10 phyla, for samples prepared in duplicate using 250 and 100 mg of bulk soil. Figure S1: Map of Virginia, USA showing the 10 sample sites from which surface soils were collected (C–H, J–M), and the land cover categorization. Figure S2: Average (± standard deviation) number of unique (*n* = 2375) and unique target sequences (*n* = 1293) recovered from samples prepared with DNA from bulk soil and individual fragments.
