# Supplementary material for: Single Fragment or Bulk Soil DNA Metabarcoding: Which is Better for Characterizing Biological Taxa Found in Surface Soils for Sample Separation?
Source: Genes (Basel). 2019 Jun 6;10(6):431. doi: 10.3390/genes10060431 (PMC6627524; doi:10.3390/genes10060431)
Supplement: Supplementary file 1 [file genes-10-00431-s001.pdf]

**Table S1.** Results summary of the wet lab DNA metabarcode protocol for DNA isolated from individual insect and plant fragments collected at 10 sites within Virginia, USA.

| Site | No. of plant fragment DNAs pooled | No. of insect fragment DNAs pooled | Amplicon Yield (ng/µL) | | | | | | Pooled Amplicon Yield (ng/µL) | Library Concentration (nM) |
|---|---|---|---|---|---|---|---|---|---|---|
| | | | *rbcL* mini | *rbcL* entire | *matK* gymno | *matK* angio | *COI* mini | *COI* entire | | |
| C | 10 | 9 | 22.6 | 27.9 | 3.3 | 26.6 | - | - | 20.0 | 21.1 |
| D | 10 | 10 | 19.6 | 32.6 | 12.1 | 30.0 | 8.5 | 4.4 | 12.8 | 15.3 |
| E | 10 | 10 | 15.0 | 36.4 | 8.6 | 16.8 | - | - | 15.4 | 21.2 |
| F | 10 | 10 | 0.3 | 0.2 | 0.3 | 0.5 | 4.8 | 9.4 | 3.8 | 8.4 |
| G | 10 | 8 | 7.8 | 8.9 | 10.2 | 1.9 | 8.8 | 20.7 | 13.2 | 22.1 |
| H | 10 | 10 | 4.4 | 9.3 | 4.5 | 2.3 | 2.5 | 6.9 | 8.8 | 9.5 |
| J | 10 | 10 | 8.5 | 10.0 | 9.6 | 1.4 | 3.6 | 0.4 | 8.7 | 13.9 |
| K | 10 | 4 | 2.8 | 5.9 | 5.5 | 0.5 | 2.6 | 2.5 | 6.3 | 8.4 |
| L | 10 | 10 | 8.4 | 22.9 | 5.6 | 9.8 | - | - | 11.7 | 14.7 |
| M | 10 | 10 | 13.6 | 15.2 | 5.9 | 13.9 | 5.4 | 0.1 | 8.2 | 13.7 |

**Table S2.** Results summary of the wet lab DNA metabarcode protocol for DNA isolated from bulk soil (either ~ 250 mg or ~100 mg) collected at 10 sites within Virginia, USA.

| Site | Bulk Soil Density ($g/cm^3$) | Soil Input Amount (mg) | DNA Yield (ng/µL) | Amplicon Yield (ng/µL) | | | | | | Pooled Amplicon Yield (ng/µL) | Library Concentration (nM) |
|---|---|---|---|---|---|---|---|---|---|---|---|
| | | | | *rbcL* mini | *rbcL* entire | *matK* gymno | *matK* angio | *COI* mini | *COI* entire | | |
| C | 1.05 | 264 | 28.4 | 26.0 | 39.0 | 5.5 | 2.4 | 16.8 | 29.6 | 37.3 | 2.1 |
| | | 214[b] | 40.6 | 30.1 | 39.7 | 3.8 | 37.2 | 11.3 | 29.4 | 46.8 | 30.0 |
| | | 98 | 31.0 | 23.6 | 40.4 | 4.3 | 4.2 | 15.3 | 30.9 | 26.2 | 32.7 |
| | | 150 | 1.2 | 5.3 | 18.0 | 0.7 | 0.6 | 16.1 | 27.3 | 0.2 | 13.5 |
| D | 1.12 | 299 | 14.5 | 23.8 | 30.2 | 3.2 | 2.0 | 17.5 | 37.3 | 24.4 | 36.5 |
| | | 217[b] | 14.2 | 17.1 | 48.6 | 3.8 | 2.6 | 25.3 | 37.2 | 36.2 | 26.7 |
| | | 90 | 14.7 | 31.2 | 21.2 | 3.0 | 27.8 | 19.0 | 36.7 | 44.3 | 39.0 |
| | | 125 | 13.9 | 21.3 | 32.5 | 1.8 | 1.0 | 17.0 | 42.6 | 36.3 | 23.2 |
| E | 0.61[a] | 270 | 1.2 | 0.9 | 0.6 | 0.5 | 0.4 | 8.8 | 23.7 | 20.0 | 36.3 |
| | | 213[b] | 6.7 | 12.0 | 21.9 | 1.2 | 10.8 | 19.9 | 41.7 | 2.0 | 28.3 |
| | | 86 | 36.4 | 20.9 | 6.8 | 2.2 | 2.7 | 16.8 | 34.6 | 21.7 | 27.5 |
| | | 148 | 34.2 | 18.8 | 13.1 | 2.2 | 1.7 | 14.6 | 34.6 | 22.5 | 17.8 |
| F | 0.51[a] | 282 | 10.5 | 1.2 | 1.5 | 0.6 | 0.9 | 3.2 | 12.1 | 5.5 | 4.2 |
| | | 221[b] | 9.8 | 2.9 | 2.7 | 1.0 | 0.7 | 0.1 | 0.2 | 1.4 | 1.4 |
| | | 88 | 9.5 | 2.7 | 2.3 | 0.9 | 0.7 | 0.9 | 11.5 | 4.5 | 4.1 |
| | | 108 | 15.1 | 1.2 | 0.8 | 0.8 | 0.7 | 0.1 | 0.1 | 1.0 | 0.8 |
| G | 0.94 | 288 | 22.0 | 4.2 | 15.2 | 1.3 | 1.1 | 10.6 | 32.5 | 22.1 | 0.2 |
| | | 207[b] | 39.1 | 11.4 | 25.1 | 2.5 | 2.5 | 7.3 | 22.6 | 16.6 | 13.0 |
| | | 96 | 20.7 | 16.0 | 17.3 | 1.5 | 2.6 | 15.4 | 39.8 | 26.1 | 25.4 |
| | | 101 | 13.7 | 10.7 | 9.0 | 0.9 | 1.2 | 7.1 | 32.1 | 21.1 | 15.2 |
| H | 1.15 | 294 | 18.0 | 15.8 | 0.6 | 3.4 | 1.6 | 17.2 | 31.0 | 29.2 | 25.3 |
| | | 203[b] | 23.0 | 23.3 | 46.1 | 13.7 | 2.3 | 18.2 | 30.0 | 31.3 | 31.3 |
| | | 126 | 3.5 | 6.2 | 17.0 | 1.3 | 0.8 | 17.1 | 30.8 | 21.1 | 16.0 |
| | | 100 | 24.5 | 17.6 | 29.8 | 11.3 | 1.2 | 17.4 | 36.0 | 29.2 | 17.6 |
| J | 0.43[a] | 262 | 0.0 | 0.6 | 0.4 | 0.5 | 0.6 | 0.1 | 0.1 | 0.5 | 0.3 |
| | | 231[b] | 0.1 | 0.5 | 0.5 | 0.5 | 0.5 | - | 0.1 | 0.6 | 0.1 |
| | | 112 | 0.1 | 0.6 | 0.6 | 0.5 | 0.6 | 0.4 | 0.1 | 0.7 | 0.7 |
| | | 98 | 0.1 | 0.5 | 0.4 | 0.4 | 0.5 | 0.1 | 0.1 | 0.6 | 0.7 |
| K | 0.8 | 298 | 37.1 | 17.1 | 32.7 | 5.6 | 4.8 | - | 36.3 | 31.5 | 19.0 |
| | | 198[b] | 40.7 | 19.7 | 37.8 | 4.7 | 3.1 | 13.7 | 25.6 | 24.6 | 27.1 |
| | | 88 | 22.4 | 18.5 | 22.5 | 2.1 | 1.8 | 16.1 | 27.3 | 36.8 | 38.7 |
| | | 133 | 19.5 | 23.7 | 30.2 | 2.4 | 2.2 | 19.2 | 32.5 | 28.7 | 19.1 |
| L | 0.85 | 282 | 11.6 | 0.627 | 10.1 | 0.965 | 1.30 | 13.8 | 31.9 | 20.5 | 35.7 |
| | | 232[b] | 17.1 | 14.9 | 19.8 | 1.47 | 2.32 | 0.216 | 0.297 | 8.67 | 21.1 |
| | | 110 | 8.68 | 3.02 | 8.30 | 0.717 | 0.736 | 0.297 | 26.1 | 18.8 | 19.3 |
| | | 97 | 13.0 | 4.38 | 9.32 | 0.795 | 0.723 | 14.2 | 35.1 | 19.4 | 0.6 |
| M | 0.72 | 300 | 24.4 | 11.6 | 34.8 | 4.17 | 2.02 | 24.5 | 40.5 | 51 | 4.2 |
| | | 210[b] | 20.1 | 23.2 | 39.1 | 4.44 | 2.74 | 28.5 | 41.2 | 37.2 | 46.6 |
| | | 86 | 18.7 | 20.8 | 34.1 | 2.10 | 1.12 | 25.6 | 31.1 | 37.8 | 39.0 |
| | | 100 | 34.5 | 15.5 | 26.6 | 2.94 | 1.41 | 20.2 | 41.9 | 32.1 | 31.0 |

[a] denotes the bulk density is indicative of a soil rich in organic matter; [b] denotes that DNA was eluted in 100 µL of Solution C6 and 4 µL of the eluate was used as input in PCR amplifications (for all other extractions, DNA was eluted in 50 µL of Solution C6 and 2 µL of the eluate was used as input in PCR amplifications); - denotes the amplicon yield was too low to be read on the Qubit Fluorometer.

**Table S3.** Average (± standard deviation) BLASTN match statistics for the best match of each unique target sequence (*n* = 1293).

| | | ***rbcL* mini (*n* = 299)** | ***rbcL* entire (*n* = 214)** | ***matK* gymno (*n* = 15)** | ***matK* angio (*n* = 20)** | ***COI* mini (*n* = 712)** | ***COI* entire (*n* = 33)** |
|---|---|---|---|---|---|---|---|
| **BLASTN matching statistics** | E-value | 0.937±1.606 | 0.522±1.366 | 2.271±2.053 | 0.998±1.398 | 0.253±0.67 | 1.025±1.723 |
| | Percent coverage | 24.6±12.5 | 24.2±29 | 29.6±23.9 | 45.2±40.8 | 40.21±25.22 | 17.2±21.2 |
| | Percent identity | 95.5±6.6 | 96.8±5.1 | 93.2±6.8 | 93.5±7.9 | 93.41±9.16 | 96±6.1 |
| | Query length | 133±39 | 236±33 | 162±95 | 206±88 | 119±42 | 235±26 |
| **Best BLASTN match status** | Green[a] | 110 | 71 | 9 | 10 | 323 | 25 |
| | Orange[b] | 15 | 28 | 5 | 1 | 42 | 0 |
| | Red[c] | 174 | 115 | 1 | 9 | 344 | 8 |

[a] denotes either a) a single record had the best match statistics, or b) there were multiple records with the same best match statistics, but all records were from the same genus and species; [b] denotes there were multiple records with the same best match statistics, but all records were from the same genus (not species); [c] denotes there were multiple records with the same best match statistics, but all records were from different genera and species.

**Table S4.** Comparison of plant taxa identified from each sample site when using DNA barcoding (i.e., Sanger) and DNA metabarcoding (i.e., MPS).

| | TAXONOMY | | | Site C | | Site D | | Site E | | Site F | | Site G | | Site H | | Site J | | Site K | | Site L | | Site M | |
|---|---|---|---|---|---|---|---|---|---|---|---|---|---|---|---|---|---|---|---|---|---|---|---|
| | *CLASS* | *ORDER* | *COMMON NAME* | *SANGER* | *MPS* | *SANGER* | *MPS* | *SANGER* | *MPS* | *SANGER* | *MPS* | *SANGER* | *MPS* | *SANGER* | *MPS* | *SANGER* | *MPS* | *SANGER* | *MPS* | *SANGER* | *MPS* | *SANGER* | *MPS* |
| ***PLANTS*** | Bryopsida | Hypnales | Moss | X | X | | | | | | | | | | | | | | | | | | |
| | Lilopsida | Poales | Grass/Reed | | | | | X | X | | | | | | | | | | | | | | |
| | Magnoliopsida | Asterales | Flowering plants | | X | | | | X | | | | | | | | | | | | | | |
| | | Brassicales | Mustards and allies | X | X | X | X | | | | | | | | | | | | | | | | |
| | | Cornales | Flowering plants | | | | | | | | | | | | | | | | | | X | | |
| | | Dipsacales | Honeysuckle | X | | | | | | | | | | | | | | | | | | | |
| | | Fagales | Oak/Stone Oak/Birch | X | | | | X | | X | | | | X | X | | | X | X | X | X | | X |
| | | Magnoliales | Magnolia | | | | | | | | | | | X | | | | | | | | | |
| | | Sapindales | Flowering plants | | | | | | | | | | | | X | | | | | | | | |
| | | Vitales | Grapevine | X | X | | | | | | | | | | | | | | | | | | |
| | Pinospsida | Pinales | Pine | X | | X | X | X | X | X | | X | X | X | X | X | X | X | X | X | X | X | X |

**Table S5.** Comparison of animal taxa identified from each sample site when using DNA barcoding (i.e., Sanger) and DNA metabarcoding (i.e., MPS).

| | TAXONOMY | | Site C | | Site D | | Site E | | Site F | | Site G | | Site H | | Site J | | Site K | | Site L | | Site M | |
|---|---|---|---|---|---|---|---|---|---|---|---|---|---|---|---|---|---|---|---|---|---|---|
| | *PHYLUM* | *CLASS* | *SANGER* | *MPS* | *SANGER* | *MPS* | *SANGER* | *MPS* | *SANGER* | *MPS* | *SANGER* | *MPS* | *SANGER* | *MPS* | *SANGER* | *MPS* | *SANGER* | *MPS* | *SANGER* | *MPS* | *SANGER* | *MPS* |
| ***ANIMALS*** | Arthropoda | Insecta | X | | X | X | | | | | | | | X | X | | | | X | | | X |
| | Chordata | Mammalia | | | | | | | | X | | | | X | | | | X | | | | X |
| | Cnidaria | Hydrozoa | | | | | | | | | | | | X | | | | | | | | |
| | Rotifera | Bdelloidea | | | | | | | | | | | | X | | | | | | | | |
| | | Monogononta | | | | | | | | X | | | | | | | | | | | | |

**Table S6.** Abundance (i.e., number of reads) of unique target sequences which met the BASTA inclusion criteria (*n* = 572) across 10 phyla, for samples prepared in duplicate using 250 mg and 100 mg of bulk soil.

| | | *Annelida* | *Arthropoda* | *Cercozoa* | *Chordata* | *Cnidaria* | *Mollusca* | *Nematoda* | *Porifera* | *Rotifera* | *Streptophyta* | TOTAL | **Total % difference** [a] |
|---|---|---|---|---|---|---|---|---|---|---|---|---|---|
| **Site C** | 250 mg - Replicate 1 | 0 | 87 | 0 | 187 | 0 | 0 | 0 | 0 | 0 | 233 | 507 | 13.5 |
| | 250 mg - Replicate 2 | 0 | 335 | 0 | 256 | 0 | 0 | 0 | 0 | 0 | 74 | 665 | |
| | 100 mg - Replicate 1 | 0 | 142 | 0 | 590 | 0 | 18 | 0 | 0 | 0 | 605 | 1355 | 31.4 |
| | 100 mg - Replicate 2 | 0 | 40 | 0 | 431 | 0 | 0 | 0 | 0 | 0 | 237 | 708 | |
| **Site D** | 250 mg - Replicate 1 | 0 | 146 | 0 | 460 | 0 | 57 | 0 | 0 | 0 | 557 | 1220 | 27.2 |
| | 250 mg - Replicate 2 | 0 | 41 | 0 | 215 | 0 | 188 | 0 | 0 | 0 | 254 | 698 | |
| | 100 mg - Replicate 1 | 0 | 119 | 0 | 94 | 0 | 0 | 0 | 0 | 0 | 72 | 285 | 30.6 |
| | 100 mg - Replicate 2 | 0 | 92 | 0 | 66 | 0 | 25 | 0 | 0 | 0 | 353 | 536 | |
| **Site E** | 250 mg - Replicate 1 | 0 | 1245 | 0 | 1151 | 0 | 3624 | 0 | 0 | 0 | 18 | 6038 | 86.6 |
| | 250 mg - Replicate 2 | 0 | 9 | 0 | 219 | 0 | 0 | 0 | 0 | 0 | 205 | 433 | |
| | 100 mg - Replicate 1 | 0 | 0 | 0 | 103 | 0 | 1591 | 10 | 0 | 0 | 788 | 2492 | 15.8 |
| | 100 mg - Replicate 2 | 0 | 0 | 0 | 0 | 0 | 2047 | 216 | 0 | 0 | 1163 | 3426 | |
| **Site F** | 250 mg - Replicate 1 | 0 | 0 | 0 | 1067 | 0 | 0 | 0 | 0 | 0 | 145 | 1212 | 67.2 |
| | 250 mg - Replicate 2 | 0 | 0 | 0 | 0 | 0 | 0 | 0 | 0 | 0 | 238 | 238 | |
| | 100 mg - Replicate 1 | 0 | 0 | 0 | 0 | 0 | 0 | 0 | 0 | 0 | 383 | 383 | 8.8 |
| | 100 mg - Replicate 2 | 0 | 0 | 0 | 0 | 0 | 0 | 0 | 0 | 0 | 321 | 321 | |
| **Site G** | 250 mg - Replicate 1 | 0 | 0 | 0 | 10 | 0 | 0 | 0 | 0 | 0 | 11 | 21 | 90.8 |
| | 250 mg - Replicate 2 | 0 | 0 | 0 | 0 | 0 | 57 | 0 | 0 | 0 | 378 | 435 | |
| | 100 mg - Replicate 1 | 0 | 322 | 0 | 167 | 0 | 75 | 0 | 0 | 0 | 260 | 824 | 15.5 |
| | 100 mg - Replicate 2 | 0 | 43 | 0 | 436 | 0 | 0 | 0 | 0 | 0 | 124 | 603 | |
| **Site H** | 250 mg - Replicate 1 | 0 | 478 | 121 | 1245 | 0 | 96 | 0 | 0 | 0 | 4076 | 6016 | 16.4 |
| | 250 mg - Replicate 2 | 31 | 77 | 0 | 510 | 0 | 0 | 0 | 0 | 0 | 7758 | 8376 | |
| | 100 mg - Replicate 1 | 0 | 163 | 46 | 916 | 0 | 42 | 0 | 0 | 0 | 2904 | 4071 | 54.4 |
| | 100 mg - Replicate 2 | 63 | 294 | 121 | 1316 | 0 | 0 | 0 | 0 | 0 | 12008 | 13802 | |
| **Site J** | 250 mg - Replicate 1 | 0 | 0 | 0 | 0 | 0 | 0 | 0 | 0 | 0 | 0 | 0 | 100.0 |
| | 250 mg - Replicate 2 | 0 | 0 | 0 | 0 | 0 | 0 | 0 | 0 | 0 | 5 | 5 | |
| | 100 mg - Replicate 1 | 0 | 0 | 0 | 0 | 0 | 0 | 0 | 0 | 0 | 0 | 0 | - |
| | 100 mg - Replicate 2 | 0 | 0 | 0 | 0 | 0 | 0 | 0 | 0 | 0 | 0 | 0 | |
| **Site K** | 250 mg - Replicate 1 | 0 | 274 | 0 | 663 | 0 | 180 | 0 | 0 | 0 | 1230 | 2347 | 16.0 |
| | 250 mg - Replicate 2 | 0 | 231 | 0 | 804 | 0 | 197 | 0 | 0 | 0 | 469 | 1701 | |
| | 100 mg - Replicate 1 | 0 | 129 | 0 | 380 | 0 | 96 | 0 | 0 | 0 | 350 | 955 | 53.6 |
| | 100 mg - Replicate 2 | 0 | 231 | 0 | 1343 | 0 | 278 | 0 | 0 | 0 | 1307 | 3159 | |
| **Site L** | 250 mg - Replicate 1 | 0 | 52 | 0 | 124 | 0 | 0 | 0 | 0 | 0 | 78 | 254 | 53.9 |
| | 250 mg - Replicate 2 | 0 | 0 | 0 | 0 | 0 | 0 | 0 | 0 | 0 | 76 | 76 | |
| | 100 mg - Replicate 1 | 0 | 13 | 0 | 355 | 0 | 26 | 0 | 0 | 0 | 83 | 477 | 64.4 |
| | 100 mg - Replicate 2 | 0 | 430 | 0 | 662 | 0 | 0 | 0 | 0 | 15 | 1093 | 2200 | |
| **Site M** | 250 mg - Replicate 1 | 0 | 369 | 0 | 609 | 0 | 0 | 0 | 7 | 0 | 328 | 1313 | 48.2 |
| | 250 mg - Replicate 2 | 0 | 52 | 0 | 194 | 0 | 0 | 0 | 2 | 0 | 211 | 459 | |
| | 100 mg - Replicate 1 | 0 | 57 | 0 | 126 | 0 | 0 | 0 | 0 | 0 | 54 | 237 | 46.5 |
| | 100 mg - Replicate 2 | 0 | 158 | 0 | 166 | 0 | 0 | 0 | 0 | 0 | 325 | 649 | |

[a] denotes that the percentage was calculated by dividing the absolute difference between replicate totals by the combined total across replicates.

**Table S7.** Number of unique target sequences which met the BASTA inclusion criteria (*n* = 572) across 10 phyla, for samples prepared in duplicate using 250 mg and 100 mg of bulk soil.

| | | *Annelida* | *Arthropoda* | *Cercozoa* | *Chordata* | *Cnidaria* | *Mollusca* | *Nematoda* | *Porifera* | *Rotifera* | *Streptophyta* | TOTAL | Total % difference [a] |
|---|---|---|---|---|---|---|---|---|---|---|---|---|---|
| **Site C** | 250 mg - Replicate 1 | 0 | 3 | 0 | 8 | 0 | 0 | 0 | 0 | 0 | 11 | 22 | 2.3 |
| | 250 mg - Replicate 2 | 0 | 4 | 0 | 8 | 0 | 0 | 0 | 0 | 0 | 9 | 21 | |
| | 100 mg - Replicate 1 | 0 | 7 | 0 | 20 | 0 | 1 | 0 | 0 | 0 | 11 | 39 | 44.4 |
| | 100 mg - Replicate 2 | 0 | 2 | 0 | 10 | 0 | 0 | 0 | 0 | 0 | 3 | 15 | |
| **Site D** | 250 mg - Replicate 1 | 0 | 2 | 0 | 9 | 0 | 1 | 0 | 0 | 0 | 11 | 23 | 21.1 |
| | 250 mg - Replicate 2 | 0 | 3 | 0 | 4 | 0 | 3 | 0 | 0 | 0 | 5 | 15 | |
| | 100 mg - Replicate 1 | 0 | 2 | 0 | 5 | 0 | 0 | 0 | 0 | 0 | 2 | 9 | 5.3 |
| | 100 mg - Replicate 2 | 0 | 2 | 0 | 3 | 0 | 1 | 0 | 0 | 0 | 4 | 10 | |
| **Site E** | 250 mg - Replicate 1 | 0 | 3 | 0 | 7 | 0 | 1 | 0 | 0 | 0 | 1 | 12 | 7.7 |
| | 250 mg - Replicate 2 | 0 | 1 | 0 | 7 | 0 | 0 | 0 | 0 | 0 | 6 | 14 | |
| | 100 mg - Replicate 1 | 0 | 0 | 0 | 4 | 0 | 1 | 1 | 0 | 0 | 10 | 16 | 8.6 |
| | 100 mg - Replicate 2 | 0 | 0 | 0 | 0 | 0 | 3 | 1 | 0 | 0 | 15 | 19 | |
| **Site F** | 250 mg - Replicate 1 | 0 | 0 | 0 | 1 | 0 | 0 | 0 | 0 | 0 | 6 | 7 | 17.6 |
| | 250 mg - Replicate 2 | 0 | 0 | 0 | 0 | 0 | 0 | 0 | 0 | 0 | 10 | 10 | |
| | 100 mg - Replicate 1 | 0 | 0 | 0 | 0 | 0 | 0 | 0 | 0 | 0 | 12 | 12 | 14.3 |
| | 100 mg - Replicate 2 | 0 | 0 | 0 | 0 | 0 | 0 | 0 | 0 | 0 | 9 | 9 | |
| **Site G** | 250 mg - Replicate 1 | 0 | 0 | 0 | 2 | 0 | 0 | 0 | 0 | 0 | 1 | 3 | 70.0 |
| | 250 mg - Replicate 2 | 0 | 0 | 0 | 0 | 0 | 1 | 0 | 0 | 0 | 16 | 17 | |
| | 100 mg - Replicate 1 | 0 | 4 | 0 | 7 | 0 | 1 | 0 | 0 | 0 | 14 | 26 | 13.0 |
| | 100 mg - Replicate 2 | 0 | 3 | 0 | 8 | 0 | 0 | 0 | 0 | 0 | 9 | 20 | |
| **Site H** | 250 mg - Replicate 1 | 0 | 5 | 1 | 9 | 0 | 1 | 0 | 0 | 0 | 24 | 40 | 5.3 |
| | 250 mg - Replicate 2 | 1 | 4 | 0 | 11 | 0 | 0 | 0 | 0 | 0 | 20 | 36 | |
| | 100 mg - Replicate 1 | 0 | 1 | 1 | 6 | 0 | 1 | 0 | 0 | 0 | 19 | 28 | 29.1 |
| | 100 mg - Replicate 2 | 1 | 6 | 1 | 14 | 0 | 0 | 0 | 0 | 0 | 29 | 51 | |
| **Site J** | 250 mg - Replicate 1 | 0 | 0 | 0 | 0 | 0 | 0 | 0 | 0 | 0 | 0 | 0 | 100.0 |
| | 250 mg - Replicate 2 | 0 | 0 | 0 | 0 | 0 | 0 | 0 | 0 | 0 | 1 | 1 | |
| | 100 mg - Replicate 1 | 0 | 0 | 0 | 0 | 0 | 0 | 0 | 0 | 0 | 0 | 0 | - |
| | 100 mg - Replicate 2 | 0 | 0 | 0 | 0 | 0 | 0 | 0 | 0 | 0 | 0 | 0 | |
| **Site K** | 250 mg - Replicate 1 | 0 | 5 | 0 | 8 | 0 | 1 | 0 | 0 | 0 | 32 | 46 | 27.8 |
| | 250 mg - Replicate 2 | 0 | 3 | 0 | 8 | 0 | 1 | 0 | 0 | 0 | 14 | 26 | |
| | 100 mg - Replicate 1 | 0 | 4 | 0 | 5 | 0 | 1 | 0 | 0 | 0 | 9 | 19 | 44.1 |
| | 100 mg - Replicate 2 | 0 | 8 | 0 | 26 | 0 | 1 | 0 | 0 | 0 | 14 | 49 | |
| **Site L** | 250 mg - Replicate 1 | 0 | 1 | 0 | 3 | 0 | 0 | 0 | 0 | 0 | 6 | 10 | 33.3 |
| | 250 mg - Replicate 2 | 0 | 0 | 0 | 0 | 0 | 0 | 0 | 0 | 0 | 5 | 5 | |
| | 100 mg - Replicate 1 | 0 | 2 | 0 | 6 | 0 | 2 | 0 | 0 | 0 | 6 | 16 | 39.6 |
| | 100 mg - Replicate 2 | 0 | 4 | 0 | 10 | 0 | 0 | 0 | 0 | 1 | 22 | 37 | |
| **Site M** | 250 mg - Replicate 1 | 0 | 1 | 0 | 7 | 0 | 0 | 0 | 1 | 0 | 8 | 17 | 21.4 |
| | 250 mg - Replicate 2 | 0 | 2 | 0 | 5 | 0 | 0 | 0 | 1 | 0 | 3 | 11 | |
| | 100 mg - Replicate 1 | 0 | 1 | 0 | 4 | 0 | 0 | 0 | 0 | 0 | 3 | 8 | 15.8 |
| | 100 mg - Replicate 2 | 0 | 3 | 0 | 3 | 0 | 0 | 0 | 0 | 0 | 5 | 11 | |

[a] denotes that the percentage was calculated by dividing the absolute difference between replicate totals by the combined total across replicates.

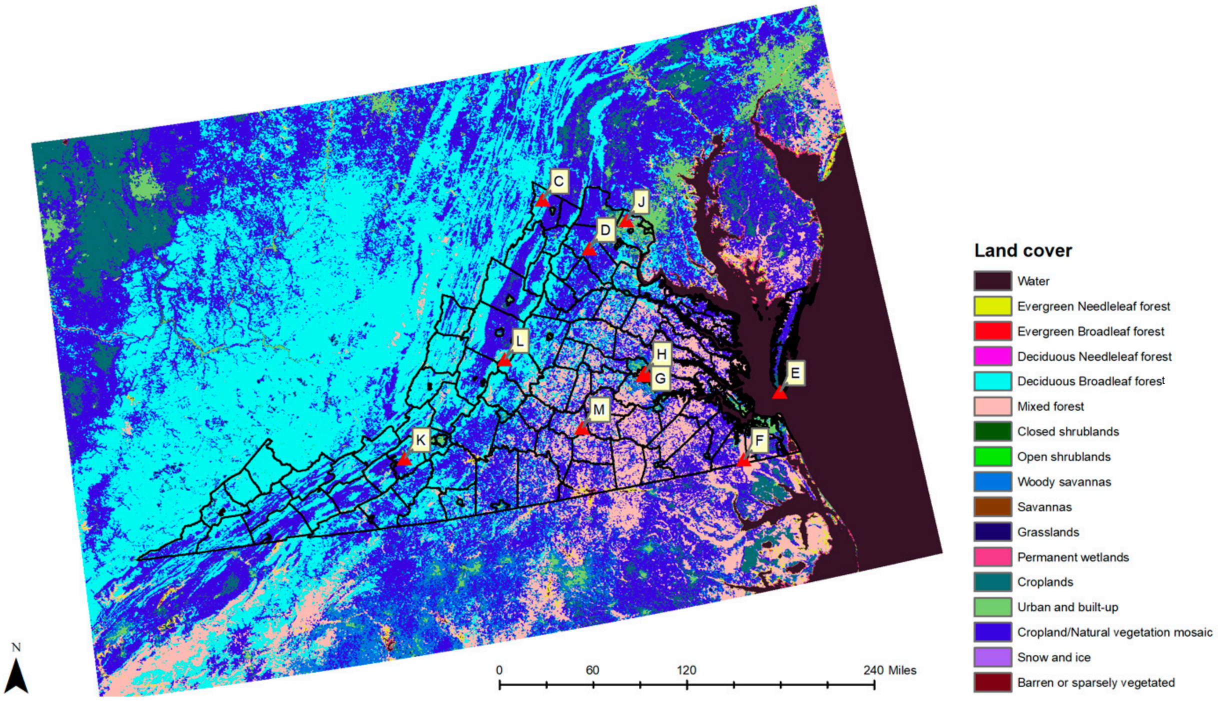


**Figure S1:** Map of Virginia, USA showing the 10 sample sites from which surface soils were collected (C–H, J–M), and the land cover categorization.

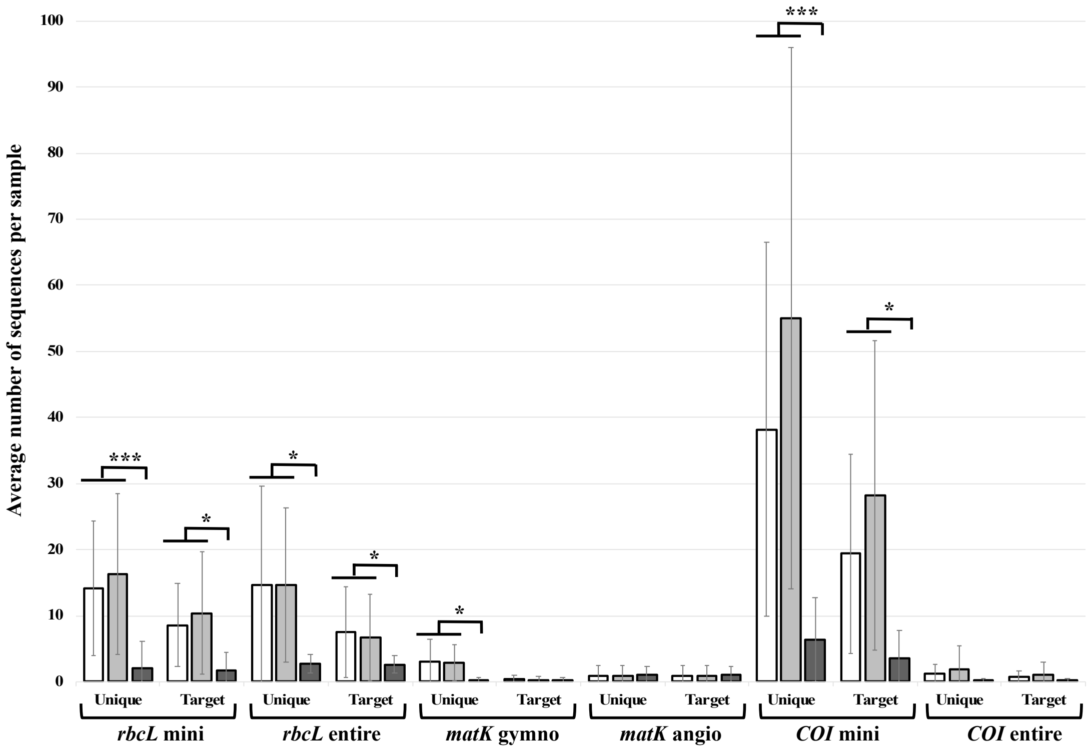


**Figure S2.** Average (± standard deviation) number of unique ($n$ = 2375) and unique target sequences ($n$ = 1293) recovered from samples prepared with 250 mg of bulk soil ($n$ = 20; denoted by white bars), 100 mg of bulk soil ($n$ = 20; denoted by light grey bars) and individual fragments ($n$ = 10; denoted by dark grey bars). Significant differences in yield (as determined by *t*-test) are shown, with * denoting $p < 0.05$ and *** denoting $p < 0.001$.
